# Comparing student outcomes in traditional vs intensive, online graduate programs in health professional education

**DOI:** 10.1186/s12909-018-1343-7

**Published:** 2018-10-20

**Authors:** Kenneth J. Harwood, Paige L. McDonald, Joan T. Butler, Daniela Drago, Karen S. Schlumpf

**Affiliations:** 1 Department of Clinical Research and Leadership, George Washington University School of Medicine and Health Sciences, 2100 Pennsylvania Ave NW, Rm 352, Washington, DC 20037-3202 USA; 2 Department of Clinical Research and Leadership, George Washington University School of Medicine and Health Sciences, 2000 W Pennsylvania Ave, NW, Rm 236, Washington, DC 20006 USA; 3 Department of Clinical Research and Leadership, George Washington University School of Medicine and Health Sciences, 2100 Pennsylvania Ave NW, Ofc 356, Washington, DC 20037-3202 USA; 4 Department of Clinical Research and Leadership, George Washington University School of Medicine and Health Sciences, 2100 Pennsylvania Ave NW, Ste 362, Washington, DC 20037-3202 USA; 5 Department of Clinical Research and Leadership, George Washington University School of Medicine and Health Sciences, 2100 Pennsylvania Ave NW, Ste 368, Washington, DC 20037-3202 USA

**Keywords:** Education, distance, Curriculum, Education, graduate, Education, professional

## Abstract

**Background:**

Health professions’ education programs are undergoing enormous changes, including increasing use of online and intensive, or time reduced, courses. Although evidence is mounting for online and intensive course formats as separate designs, literature investigating online and intensive formats in health professional education is lacking. The purpose of the study was to compare student outcomes (final grades and course evaluation ratings) for equivalent courses in semester long (15-week) versus intensive (7-week) online formats in graduate health sciences courses.

**Methods:**

This retrospective, observational study compared satisfaction and performance scores of students enrolled in three graduate health sciences programs in a large, urban US university. Descriptive statistics, chi square analysis, and independent t-tests were used to describe student samples and determine differences in student satisfaction and performance.

**Results:**

The results demonstrated no significant differences for four applicable items on the final student course evaluations (*p* values range from 0.127 to 1.00) between semester long and intensive course formats. Similarly, student performance scores for final assignment and final grades showed no significant differences (*p* = 0.35 and 0.690 respectively) between semester long and intensive course formats.

**Conclusion:**

Findings from this study suggest that 7-week and 15-week online courses can be equally effective with regard to student satisfaction and performance outcomes. While further study is recommended, academic programs should consider intensive online course formats as an alternative to semester long online course formats.

## Background

Health professions’ education programs are undergoing enormous changes that are, in part, reactions to changes in students’ preferences and demographics as well as to increasing technological advances. Recent evidence suggests that current adult students expect flexibility in the delivery mode and structure of undergraduate and graduate education [1–3]. These expectations include the use of online delivery models and intensive course structures. These expectations also impact health and medical professional education, as online and intensive courses (ICs) are increasingly being implemented in various health professions’ curricula [4–6].

Recently, student registration in online courses has significantly increased. In 2015, 29.7% of all students in US higher education were taking at least one distance education course, representing a 3.9% increase from the previous year [7]. Research noting the advantages of online education explains this increase. Adult learners enroll in online programs for increased accessibility, flexibility in delivery mode, and self-direction in the process of learning [8]. Within health and medical professional education, authors confirm increased accessibility, flexibility and self-direction as benefits of online learning, but also note additional benefits such as increased interactivity among participants and improved cost [9–11]. With regard to learning in health professions and medical education, Cook et al. [12] conducted a systematic review investigating studies on the effects of online instruction and learning compared to studies with no online component. The authors found a large positive effect of online courses compared to no instruction and similar effectiveness compared to more traditional delivery methods. Reis et al. [13] investigated 40 medical students who learned urology content in either a face-to-face lecture format or student centered group discussions in a 4-week online course using the Moodle platform (modular object-oriented dynamic learning environment). The results demonstrated that 86% of the students thought the online course was superior to the face-to-face delivery method. Specifically, the online content was better in “encouraging and motivating learning,” “arousing interest in the topic,” and “fostering teacher and student interactions” (pg. 152). Interestingly, the authors found a smaller range of grades on the final “increment in learning” assessment for the students in the online format (7.0–9.7) as compared to the face-to-face delivery format (4.0–9.6).

In adopting online delivery models, institutions of higher education have also been condensing the delivery time of courses, both online and face-to-face. ICs have been increasingly adopted in institutes of higher education [14–16]. ICs are defined as courses which deliver the amount of content typically presented in a traditional 15 or 16-week semester in an intensive (time reduced) period [15, 17–19]. Fanjoy [20] reported that course offerings of online ICs increased from 22 to 36% for 67 public, four-year institutions between 2007 and 2008 for the summer semesters. Like online learning, ICs appeal to the growing number of non-traditional students who have difficulty meeting the demands of courses more traditional in length or delivery method [8, 14]. These non-traditional students tend to be “slightly older and working students, with slightly higher GPAs than students in traditional courses” ([16], p.1109). Literature suggests that non-traditional students prefer ICs due to convenience [14], efficient use of student time [21], and shorter time to completion [22]. In addition, faculty and students think ICs promote a “continuous learning experience” that enables a more intense connection to the content because students focus on fewer classes at one time [14].

Still, higher education research suggests drawbacks to IC course structure and inconclusive evidence regarding overall effectiveness and student satisfaction. ICs require a more concentrated effort in a shorter amount of time, thus reducing the time for students to review and learn course material and complete assignments [14, 23]. In addition, some researchers suggest this shortened time-frame may be related to the increases in student reported stress associated to the IC as compared to traditional length courses [14, 15, 23, 24]. Further, studies comparing IC to semester long courses remains inconclusive. Kucsera and Zimmaro [18] report no significant difference in instructor ratings between online ICs and semester long courses. In reviewing the literature, Hall et al. [16] suggest that a majority of investigations comparing ICs to courses of traditional length demonstrate that ICs are associated more with student success; however, a significant proportion of other studies show no difference. Results on students’ satisfaction are also mixed. Wlodkowski et al. [25] report that students’ overall attitudes toward ICs were positive in comparison to semester long courses. Whillier et al. [26] note equivalent findings regarding student satisfaction; whereas, Mishra et al. [23] find students mostly dissatisfied with ICs. It is important to note an absence of comparative research on ICs delivered online versus semester long courses delivered online.

When considering adoption of ICs in health professions education, Sonnadara and colleagues [27] found that a face-to-face IC at the beginning of a first year orthopedic residency was “highly effective” in teaching targeted surgical skills. On the other hand, Whillier and Lystad [26] concluded that a cohort of students who were involved in a semester long course attained significantly higher final grades compared to a cohort taught the same content in an IC. Regarding test scores, some evidence reports comparable results between IC and semester long courses [14] and some report slightly higher results for ICs [28, 29]. Yet, Petrowsky [30] found students in ICs performed worse on comprehensive examinations. However, as of date, there is an absence of research comparing online ICs and semester long courses, in either an online or face-to-face delivery model, in health professions education.

As online health professional programs consider transitioning from a semester long course model to an IC format, further research is necessary to clarify the effects of transitioning on student performance and satisfaction. Hence, the purpose of this paper is to compare student outcomes (performance and satisfaction) in equivalent, graduate-level, health science courses offered in a 7-week intensive (IC) online format and 15-week semester long online format.

## Methods

### Study aims

The aims of the study were:

1. To compare student course evaluation scores for equivalent courses in semester long versus intensive formats in online, graduate level health sciences courses.
2. To compare student performance scores for equivalent courses in semester long versus intensive formats in online, graduate level health sciences courses.

### Study design

This was a retrospective, observational study. A convenience sample was selected from three health sciences graduate programs at George Washington University, School of Medicine and Health Sciences, a large urban US university. Each program offers an 18 credit graduate certificate and a 36 credit Masters of Science in Health Sciences (MSHS) degree. Table 1 describes each program briefly.

**Table 1:** Health Sciences Graduate Program Descriptions

Program	Program Description
Clinical Research Administration	The Clinical Research Administration program prepares health sciences professionals to participate in the science and business of developing new therapeutics for improving patient care. The rigorous curriculum focuses on regulatory requirements, ethical issues, processes for product development, the business of clinical research, and scientific method processes for product development.
Health Care Quality	The Health Care Quality program incorporates an interdisciplinary, practice-based curriculum to prepare individuals with healthcare experience for leadership roles in quality-based healthcare. Graduates of this program gain the knowledge and skills necessary to redefine quality care, including healthcare leadership and organizational change theories and principles, collaboration and safe practices within healthcare teams, risk assessment in patient safety systems, navigating environmental changes affecting the healthcare enterprise, and health policy development, implementation and measurement.
Regulatory Affairs	Developed in collaboration with regulatory affairs professionals in governmental agencies, including the Food and Drug Administration (FDA) and the National Institutes for Health (NIH), the Regulatory Affairs program integrates global regulatory strategy across the curriculum to equip graduates as business leaders in regulatory strategy locally and abroad. The program content focuses on clinical research, product testing, global health, and public health policy.

Each program underwent separate but related processes to condense the 15-week semester long curricula to a 7-week intensive format. The curricular changes were coordinated among programs through a steering committee; however, individual programmatic changes were allowed to meet the program’s student outcomes and curricular needs. Students were transitioned into the new structure as they entered the program, so they did not select the curriculum format. The semester long and IC versions of the programs used asynchronous teaching methods. The IC courses were designed to include student cohorts with students taking one course at a time using a rigid sequence of courses and greater use of online technology, however the 15 week programs allowed students to take two courses a semester with greater flexibility in course sequencing. The detail of the associated changes to pedagogy and curriculum are more fully described in a companion publication [31]. The Institutional Review Board approved this study as exempt.

### Course selection

To compare the effects of the curricula changes, courses within each program were selected for comparison based on two inclusion criteria. First, the course instructor was the same for each version of the course (i.e., 7- and 15-weeks). Second, the 7-week and 15-week versions of each course had the same or similar learning objectives, course content, and final assessment. These criteria were applied to control for potential differences in faculty instruction and course assignments. The final assessment in each course was a written, evidence-based paper. A total of seven pairs of courses were selected for comparison – one pair from the clinical research administration (CRA) program, two from health care quality (HCQ), and three from regulatory affairs (RAFF). In addition, one health sciences core (HSCI) course offered in all three programs was included in the analysis. Health sciences courses are foundational graduate courses that are shared among programs (i.e. biostatistics, epidemiology, leadership).

### Subjects

The sample included graduate or graduate certificate student records in three programs of study, clinical research administration (CRA), health care quality (HCQ), and regulatory affairs (RAFF). As is typical with these programs, a majority of students were adults who maintained full or part time employment in health care or related fields during matriculation.

### Assessments

The courses were compared on measures of student satisfaction and student performance. Student satisfaction was assessed using scores from course evaluations completed through a voluntary, online university system at end of the class. Student satisfaction assessment was based on four items from the university-developed standardized course evaluation form, including: (1) “overall rating of the course,” (2) “how much they learned in the course,” (3) “intellectual challenge,” and (4) “overall instructor rating.” Each item was rated on a 5-point Likert scale. Student performance in the course was assessed in two ways: (1) the final assignment, which in most cases was a summative, evidence-based paper; and (2) the course final grade. Letter grades were reported as the equivalent mean numerical percentage (e.g., an A- was reported as 91.5%).

### Data analysis

Student satisfaction and student performance data from each course were de-identified and coded by program administrative staff and provided to the researchers. Administrative staff also provided additional de-identified student data regarding age, program of study, credit hours completed, and cumulative GPA. All data analyses including sample summary and comparative statistics were performed using SAS (version 9.3, SAS Institute Inc., Cary NC, USA) and SPSS (version 24, IBM Corp., Armonk, N.Y., USA).

Descriptive statistics were generated to describe the study population, student satisfaction scores, and course grades. Chi-square analyses were conducted to compare student satisfaction scores between 7-week intensive and 15-week semester long courses. Ratings were dichotomized to “High” or very favourable (4 or 5) and “Low” or less favourable (1–3) for comparison purposes. To determine differences in the final assignment and final course grades between the two course formats, independent t-tests were run. All inferential statistics were conducted first for an overall comparison between 7-week and 15-week formats for all courses in total and second for a comparison by course.

## Results

The study assessed 245 health sciences student records of which 35.1% were enrolled in the clinical research administration program (CRA), 42.9% were enrolled in the health care quality program (HCQ), and 22.0% were enrolled in the regulatory affairs program (RAFF). The study population’s mean total credit hours completed was 28.3 (range: 3–60) credits indicating that most were nearing the end of the programs of study. The mean cumulative GPA of the study population was 3.57 (range: 0–4.00).

**Fig. 1:**
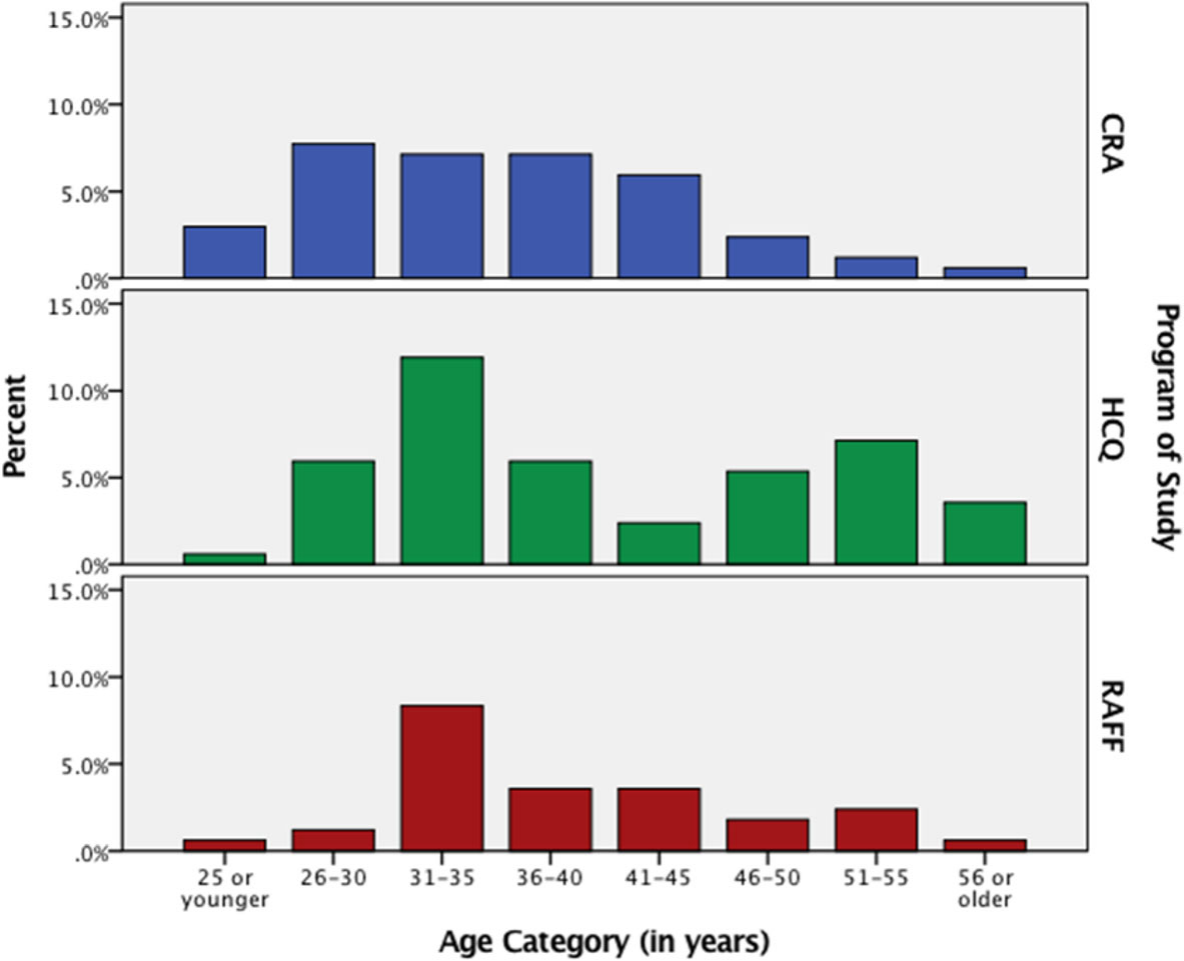
Bar graph of percent (%) of students’ shown by age category (years) for the programs

Approximately 44% of the students in this study were between 31 to 40 years of age. Figure 1 shows the distribution of age categories for the total study population by program. Most students enrolled in the CRA program are between 26 and 40 years of age (62.6%). Within the HCQ program, 27.8% of the students are between the ages of 31 and 35 with a second peak of 16.7% of the students between the ages of 51 and 55. Almost 40% of RAFF students are between 31 to 35 years of age.

### Student satisfaction

Ninety-nine students in ICs and seventy-seven students in 15-week format completed the end of course evaluations. It is important to note that the course evaluations are voluntary, so the total numbers of students’ responses will vary depending upon the courses, the survey question, and student interest. The course evaluation response rate varied between 20 and 100% of eligible respondents over all courses (Appendix). In the overall comparison of student satisfaction, results from the chi-square analysis indicated no significant differences in ratings between 7-week and 15-week formats for all four items in the course evaluations (Table 2). Comparisons by each course yield similar findings except for the Health Care Quality Course #2 (Appendix). Students in the intensive 7-week curricula reported significantly lower satisfaction course evaluation ratings (i.e. 1–3) for both intellectual challenge (*p* = 0.033) and instructor rating (*p* = 0.026).

**Table 2:** Chi-Square results for student course evaluation ratings

		Rating		*P*-value
		Low n (%)	High n (0%)	
Overall Course Rating	Intensive Traditional	14 (15.6) 11 (15.5)	76 (84.4) 60 (84.5)	1.000
How Much Learned	Intensive Traditional	11 (11.1) 6 (8.5)	88 (88.9) 65 (91.5)	0.615
Intellectual Challenge	Intensive Traditional	18 (18.9) 7 (9.9)	77 (81.1) 64 (90.1)	0.127
Overall Instructor Rating	Intensive Traditional	12 (12.4) 4 (5.6)	85 (87.6) 67 (94.4)	0.186

### Student performance

A total of 245 student grades (final grade assignment and final grade) were analyzed from the three programs in both the 15-week and 7-week versions of the courses, including 136 enrolled in the ICs and 109 in the semester long courses. Results of the overall comparisons in student performance measures indicate no significant differences between intensive and semester long course formats (Table 3). In addition, no significant differences were found between IC and semester format in final grade and final assignment for each course (Table 4 & Table 5). Differences in mean final assignment and course grades were small.

**Table 3:** Independent t-Test results for student performance

				P-value
		N	Mean (SD)	
Final Assignment	Intensive Traditional	136 109	91.5 (7.6) 89.8 (17.4)	0.353
Final Grade	Intensive Traditional	136 109	91.1 (5.8) 91.5 (9.3)	0.690

**Table 4:** Final assignment grade comparison 7-week versus 15-week (by course)

	Course Structure	N	Mean (SD)	t-score	df	P-value
CRA Course 1	Intensive	35	86.2 (8.3)	−0.084	54	0.934
	Traditional	21	86.4 (12.6)			
HCQ Course 1	Intensive	12	96.2 (5.8)	0.953	43	0.346
	Traditional	33	91.2 (17.9)			
HCQ Course 2	Intensive	37	94.6 (6.8)	−0.798	58	0.428
	Traditional	23	96.0 (6.5)			
RAFF Course 1	Intensive	8	88.9 (4.8)	0.895	13	0.387
	Traditional	7	77.8 (34.8)			
RAFF Course 2	Intensive	13	92.8 (4.7)	0.365	19	0.719
	Traditional	8	92.1 (4.9)			
RAFF Course 3	Intensive	10	83.5 (4.7)	0.908	4.1	0.414
	Traditional	5	68.0 (38.0)			
HSCI Course 1	Intensive	21	96.0 (1.5)	1.281	13.7	0.221
	Traditional	12	94.8 (3.3)

**Table 5:** Final course grade comparison 7-week versus 15-week (by course)

	Course Structure	N	Mean (SD)	t-score	df	*P*-value
CRA Course 1	Intensive	35	87.7 (5.3)	−0.005	24.7	0.996
	Traditional	21	87.7 (12.1)			
HCQ Course 1	Intensive	12	94.5 (3.9)	0.340	43	0.736
	Traditional	33	93.6 (8.0)			
HCQ Course 2	Intensive	37	92.5 (5.1)	−0.807	58	0.423
	Traditional	23	93.5 (4.4)			
RAFF Course 1	Intensive	8	89.9 (5.2)	0.770	13	0.455
	Traditional	7	85.6 (14.8)			
RAFF Course 2	Intensive	13	90.6 (3.5)	−0.710	19	0.486
	Traditional	8	91.8 (4.4)			
RAFF Course 3	Intensive	10	84.6 (4.0)	0.266	4.2	0.803
	Traditional	5	82.6 (16.2)			
HSCI Course 1	Intensive	21	96.3 (1.1)	1.456	14	0.167
	Traditional	12	95.3 (2.3)			

## Discussion

While previous studies have compared the effectiveness of semester long courses and ICs delivered in face-to-face contexts with inconclusive results, this study is unique in that it considered the effectiveness of 15-week and 7-week format for online courses. While the results of prior research related to student satisfaction with face-to-face ICs and face-to-face semester long courses were mixed [1–4] this study indicates no significant difference in student satisfaction between ICs and semester long courses delivered online. This research confirms the Kucsera and Zimmaro [18] findings of no significant difference in instructor ratings and the Whillier and Lystad [26] findings of no significant difference in overall student satisfaction. As higher education and health professions’ education seek to identify learning models which meet the needs of a wider range of students, including non-traditional, working adult learners, the findings support that adoption of IC models for online courses as a viable choice.

The results of the study confirm previous findings where no significant difference in student success was found between ICs and semester long courses [6] and disconfirms Whillier and Lystad’s [26]) findings that semester long course formats yield higher student success. As noted in our companion paper [31], our team adopted a very structured process for curriculum re-design when transitioning from a semester long to IC delivery model. This process and our corresponding focus on the alignment between course objectives and course assignments may help explain these findings. The controlled sequencing of courses in the IC format, which allowed for scaffolding of knowledge across courses, may also help explain these findings. For other programs of study seeking to re-design programs to optimize space and time in learning delivery, these results suggest that online ICs can be a comparable choice to 15-week extended models of delivery, particularly if emphasis in re-design is placed upon re-alignment of content to course objectives, rather than to merely condensing existing content, and sequencing courses to scaffold knowledge essential to achieving program competencies.

While the findings indicate no significant difference in student performance and satisfaction, it is important to consider the limitations of this study and what they suggest for future research. Regarding sampling, the selection of a convenience sample from three health sciences graduate programs may introduce selection bias. Regarding student satisfaction, we identified four items from the end of course evaluations to characterize “satisfaction” (i.e., overall course rating, how much learned, intellectual challenge, and instructor rating). However, definitions for these items are not provided on the evaluations; therefore, it cannot be assumed that all students interpreted the meaning of these items in the same way. In addition, course evaluations – particularly for courses with low response rates – may not present an accurate assessment of the quality of a course, especially as results may be skewed by a respondent who has an axe to grind.

Other potential limitations relate to the comparison of final assignment grades and final course grades. Final qualitative paper grades were used within this study rather than didactic tests. These types of assessments (papers) were thought to align more readily with determining achievement of course objectives; however, they are less reliable than didactic tests because they may introduce bias in grading. In addition, it is possible that there was a ceiling effect as the range of the final grades and assignments were in the “A” to “A-”range. To counteract some of the bias, rubrics for grading final assignments were used. Also, bias was mitigated by comparing courses taught by the same instructors. With regard to final course grades, grades assigned by instructors within each course varied between letter and numerical grades. Although we applied a mean numerical value to represent letter grades, our estimates of the differences in final assignment and course grades may not detect small variations in grades.

## Conclusions

Findings from this study suggest that 7-week and 15-week online courses can be equally effective with regard to student satisfaction and performance outcomes. However, additional research is required as health professions education and higher education, wrestle with selecting delivery models that align both with the needs of the learner and with the needs of the faculty and institutions. In particular, additional research is needed on the faculty experience of teaching 7-week versus 15-week courses, particularly in online contexts. Variables to consider in this research are the number of courses faculty have previously taught in each delivery model and how this number might influence their experience in teaching. Also, faculty workload in facilitation in different delivery models should be considered. For future comparative educational effectiveness studies on online models of delivery, research must also consider faculty experience in online facilitation and how it might influence course evaluations, facilitation style, and grades.

Finally, additional longitudinal research is required to determine the long term effects of different delivery models on overall performance, satisfaction (both student and faculty), and retention of knowledge over time. Future research might also consider different methodological approaches, such as mixed methods, by which to assess the comparative quality of courses delivered in different models. With regard to online ICs, longitudinal research across different programs of study would allow greater understanding of the variables that influence facilitation and learning across different course content.

## Appendix

### Student Course Evaluation Ratings by Course

**Table 6:** Chi-square results for evaluation item on “Overall course rating” (by course)

		Course Enrollment	Respondents	Response Rate (%)	Rating		*P*-value
					Low n (%)	High n (%)	
CRA Course 1	Accelerated Traditional	45 23	38 16	84.4 69.6	2 (5.3) 2 (12.5)	36 (94.7) 14 (87.5)	0.573
HCQ Course 1	Accelerated Traditional	12 12	6 6	50.0 50.0	3 (50.0) 4 (66.7)	3 (50.0) 2 (33.3)	1.000
HCQ Course 2	Accelerated Traditional	38 44	23 29	60.5 65.9	6 (26.1) 2 (6.9)	17 (73.9) 27 (93.1)	0.118
RAFF Course 1	Accelerated Traditional	9 7	9 6	100.0 85.7	1 (11.1) 0 (0.0)	8 (88.9) 6 (100.0)	1.000
RAFF Course 3	Accelerated Traditional	10 5	4 1	40.0 20.0	2 (50.0) 1 (100.0)	2 (50.0) 0 (0.0)	1.000
HSCI Course 1	Accelerated Traditional	21 13	10 7	47.6 53.8	0 (0.0) 1 (14.3)	10 (100.0) 6 (85.7)	0.412

**Table 7:** Chi-square results for evaluation item on “How much learned” (by course)

		Course Enrollment	Respondents	ResponseRate	Rating		*P*-value
					Low n (%)	High n (%)	
CRA Course 1	Accelerated Traditional	45 23	38 16	84.4 69.6	2 (5.3) 1 (6.3)	36 (94.7) 15 (93.8)	1.000
HCQ Course 1	Accelerated Traditional	12 12	6 6	50.0 50.0	3 (50.0) 2 (33.3)	3 (50.0) 4 (66.7)	1.000
HCQ Course 2	Accelerated Traditional	38 44	23 29	60.5 65.9	3 (13.0) 0 (0.0)	20 (87.0) 29 (100.0)	0.080
RAFF Course 1	Accelerated Traditional	9 7	9 6	100.0 85.7	1 (11.1) 0 (0.0)	8 (88.9) 6 (100.0)	1.000
RAFF Course 3	Accelerated Traditional	10 5	5 1	50.0 20.0	0 (0.0) 1 (100.0)	5 (100.0) 0 (0.0)	0.167
HSCI Course 1	Accelerated Traditional	21 13	18 7	85.7 53.8	2 (11.1) 1 (14.3)	16 (88.9) 6 (85.7)	1.000

**Table 8:** Chi-square results for evaluation item on “Intellectual challenge” (by course)

		CourseEnrollment	Respondents	Response Rate	Rating		*P*-value
					Low n (%)	High n (%)	
CRA Course 1	Accelerated Traditional	45 23	34 16	75.6 69.6	5 (14.7) 1 (6.3)	29 (85.3) 15 (93.8)	0.650
HCQ Course 1	Accelerated Traditional	12 12	6 6	50.0 50.0	3 (50.0) 3 (50.0)	3 (50.0) 3 (50.0)	1.000
HCQ Course 2	Accelerated Traditional	38 44	23 29	60.5 65.9	4 (17.4) 0 (0.0)	19 (82.6) 29 (100.0)	*0.033*
RAFF Course 1	Accelerated Traditional	9 7	9 6	100.0 85.7	2 (22.2) 0 (0.0)	7 (77.8) 6 (100.0)	0.486
RAFF Course 3	Accelerated Traditional	10 5	5 1	50.0 20.0	1 (20.0) 1 (100.0)	4 (80.0) 0 (0.0)	0.333
HSCI Course 1	Accelerated Traditional	21 13	18 7	85.7 53.8	3 (16.7) 1 (14.3)	15 (83.3) 6 (85.7)	1.000

**Table 9:** Chi-square results for evaluation item on “Overall instructor rating” (by course)

		Course Enrollment	Respondents	Response Rate	Rating		*P*-value
					Low n (%)	High n (%)	
CRA Course 1	Accelerated Traditional	45 23	38 16	84.4 69.6	1 (2.6) 0 (0.0)	37 (97.4) 16 (100.0)	1.000
HCQ Course 1	Accelerated Traditional	12 12	6 6	50.0 50.0	3 (50.0) 2 (33.3)	3 (50.0) 4 (66.7)	1.000
HCQ Course 2	Accelerated Traditional	38 44	21 29	55.3 65.9	4 (19.0) 0 (0.0)	17 (81.0) 29 (100.0)	*0.026*
RAFF Course 1	Accelerated Traditional	9 7	9 6	100.0 85.7	0 (0.0) 0 (0.0)	9 (100.0) 6 (100.0)	NA
RAFF Course 3	Accelerated Traditional	10 5	6 1	60.0 20.0	2 (40.0) 1 (100.0)	4 (60.0) 0 (0.0)	1.000
HSCI Course 1	Accelerated Traditional	21 13	18 7	85.7 53.8	2 (11.1) 0 (0.0)	16 (88.9) 7 (100.0)	1.000
